# Refractory periods in SUNCT

**DOI:** 10.1007/s10194-012-0463-4

**Published:** 2012-06-05

**Authors:** Juan A. Pareja, Mónica Álvarez, Teresa Montojo

**Affiliations:** Department of Neurology, University Hospital Fundación Alcorcón, Budapest 1, 28922 Alcorcón, Madrid Spain

Dear Sir,

The article by Paliwal et al. [1] brings to mind the importance of assessing the presence/absence of refractory periods in the differential diagnosis of short-lasting unilateral neuralgiform headache with conjunctival injection and tearing (SUNCT), primarily because trigeminal neuralgia attacks are systematically followed by refractory periods, contrary to what is frequent in SUNCT.

Since the first communication of SUNCT [2], it has been known that post-attack refractory periods may be only relative or completely absent. Moreover, we reported that SUNCT paroxysms could be triggered during the “descrescendo” phase of an ongoing attack [3], and that occasionally, the usual stimuli seemed totally ineffective, even when applied for a prolonged time [4]. The sensitivity, or threshold, for attack generation, may therefore, differ from time to time. Later, we documented these findings [5] in a patient in whom many SUNCT attacks could be modulated by lengthening of the trigger stimuli.

Indeed, we first described the four patterns of the temporal profile of solitary SUNCT attacks: “plateau-like”, “saw-tooth like”, “repetitive”, and “plateau-like plus exacerbations” [4]. A repetitive pattern of sharp or spike-like paroxysms, without reaching the baseline in between the solitary peaks, inherently carried important pathophysiological implications, as spontaneous or triggered attacks overlapped each other, thus substantiating our observations that SUNCT attacks may lack refractory periods.

Available knowledge regarding both quantitative and qualitative temporal aspects of SUNCT attacks has been extensively reported by our group [2–5]. Unfortunately, Paliwal et al. [1] did not mention our early findings and mistakenly referenced the absence of refractory periods in SUNCT to an article by Cohen et al. [6]. For the sake of accuracy it should be noted that, in regards to this subject, Cohen et al. simply replicated our findings, many years later.
